# Symptomatology and Demographic Profile of COVID-19 Patients Admitted to a Tertiary Care Center in India: A Hospital Record-Based Study

**DOI:** 10.7759/cureus.40636

**Published:** 2023-06-19

**Authors:** Arumadi Anupama, Rupesh Raman, R. Ratheesh, Sujesh Palakkunnath

**Affiliations:** 1 Community Medicine, Kunhitharuvai Memorial Charitable Trust (KMCT) Medical College, Kozhikode, IND; 2 Pharmacology, Kunhitharuvai Memorial Charitable Trust (KMCT) Medical College, Kozhikode, IND

**Keywords:** sars-cov-2, india, kerala, symptoms, epidemiology, covid-19

## Abstract

Introduction: Since its arrival in late 2019, COVID-19 has caused more than 760 million cases and nearly seven million deaths worldwide. As a novel infection research is still underway to understand the epidemiology of COVID-19. The present study was conducted in a tertiary care center in south India to understand the symptomatology of the disease in a local context.

Methods: Information for the study was retrieved from the hospital records of the Kunhitharuvai Memorial Charitable Trust (KMCT) COVID Hospital, Kerala, India, of patients admitted from 1st May 2021 to 31st October 2021 (six months). Data on their clinico-demographic profile and treatment outcomes were collected and entered into a proforma.

Results: Out of the 2744 patients included in the study, the proportion of males and females was comparable. About 38.6% of patients were above 60 years of age. The most common presenting complaints were fever, cough, and breathlessness. About 2.7% were asymptomatic. The mortality rate during the study period was 4.8% (132 deaths).

Conclusion: The present study highlights differences in the symptomology and other demographic features of COVID-19 patients admitted to a hospital in Kerala, India, compared to other regional, national, and international studies. Despite limitations, these differences may have important implications for diagnosing and managing COVID-19 patients in the region.

## Introduction

As the world is learning to live with COVID-19, it is becoming increasingly clear that this pandemic has caused profound changes in nearly every aspect of human life, including social, economic, and political systems. From widespread lockdowns and remote work to mask mandates and social distancing, the pandemic has forced individuals and societies to adapt to a “new normal”. Since its arrival in late 2019, COVID-19 has caused more than 760 million cases and nearly seven million deaths worldwide [1]. In India, there have been more than 44 million cases and more than 530 thousand deaths, with the southern state of Kerala accounting for about 6.7 million cases and 70 thousand deaths [1,2].

The spectrum of the COVID-19 disease ranges from asymptomatic to critical illness, with fever being one of the most common manifestations of the disease [3]. Dry cough, anorexia, hyposmia, myalgia, dyspnea, and gastrointestinal symptoms like diarrhea and abdominal pain have also been reported. Although the disease can affect any age group, the elderly and patients with comorbidities are at higher risk for severe disease [4].

As a new disease, research is still underway to understand the epidemiology of COVID-19. The objectives of our study are to explore the symptomatology and demographic profile of lab-confirmed cases of COVID-19. It aims to help shed more light on our current understanding of the disease and help tackle future outbreaks in a more efficient manner.

## Materials and methods

We report findings from a designated 450‑bedded COVID hospital in Kerala, a southern state in India. The hospital received patients mainly from Kozhikode district and the adjoining districts of Malappuram and Wayanad. The hospital provided services including testing, triage, treatment including chest physiotherapy and superspeciality consultation, discharge, and follow-up. A descriptive cross-sectional study design has been used for the present study.

Patients arriving at the hospital were received in the triage area of the hospital. Their vital signs as well as oxygen saturation were recorded, and basic blood investigations were sent. Inflammation markers like CRP and D-dimer were sent as indicated by clinical severity. Patients were categorized and managed according to the Kerala state treatment guidelines [5]. All staff were given adequate training on the management of the patients as per the guidelines and were intimated promptly in case of any updates or reviews of the same.

All the patients received symptomatic support as well as treatment for any pre-existing or newly detected comorbidities. Oxygen support was immediately made available to patients with saturation less than 94% with the help of a nasal prong, a face mask and a non-rebreathing mask as clinically indicated.

The state guidelines were followed for the discharge of the patients as well [6]. Test negativity has not been mandatory for patients with mild and moderate disease since 25th April 2021. Only patients with severe disease were required to be antigen-negative for discharge during the study period. If the test was positive, it was repeated every 48 hours until the patient became negative. Upon discharge, all patients were advised for follow-up in the post-COVID clinic of the hospital.

Information for the study was retrieved from the hospital records of the Kunhitharuvai Memorial Charitable Trust (KMCT) COVID hospital of patients admitted from 1st May 2021 to 31st October 2021 (for a period of six months), which were stored in the hospital database. A total of 2744 patients admitted during this study period were included in the study with no exclusions. Data on their clinico-demographic profile and treatment outcomes were collected and entered into a proforma, which contained variables like name, age, sex, symptoms, treatment outcome, etc., which were then entered into a Microsoft Excel sheet (Microsoft Corporation, Redmond, USA). Mortality during this study period was also calculated.

Statistical analysis

Statistical Package for the Social Sciences (SPSS, IBM SPSS Statistics for Windows, Trial Version 26.0., IBM Corp., Armonk, NY) was used for statistical analysis. Continuous data are expressed as means and standard deviations, and categorical data are expressed as frequencies and percentages.

Ethical considerations

The study was approved by the Institutional Ethics Committee, KMCT Medical College, Approval No. KMCT/MC/IRC/10331122021. The confidentiality of the data, as well as patient details, was maintained throughout the study.

## Results

A total of 2744 patients admitted during the study period (1st May 2021-31st October 2021) were included in the study. The median age of the study population was found to be 55 years with a range of six days to 100 years. About 1412 (51.5%) of the participants were female. The demographic profile of the participants is shown in Table 1.

**Table 1:** Demographic profile of COVID-19 patients admitted to the hospital (n=2744).

	Frequency	Percentage
Gender		
Males	1332	48.5
Females	1412	51.5
Age group		
0-20 Years	123	4.5
21-40 Years	552	20.12
41-60 Years	1010	36.81
>60 Years	1059	38.60

The most common presenting symptom was fever (44.56%), followed by cough (41.1%). Breathlessness was also seen in 29.5% of the patients. Other symptoms like headache, fatigue, and diarrhea were also reported. The profile of symptoms in the patients is shown in Table 2.

**Table 2:** Profile of symptoms of patients (n=2744).

Symptoms	Frequency	Percentage
Fever	1223	44.56
Cough	1129	41.1
Breathlessness	810	29.5
Fatigue	598	21.8
Headache	527	19.2
Gastrointestinal symptoms	398	14.5
Myalgia	286	10.4
Sore throat	163	5.9
Chest discomfort	154	5.6
Rhinitis	65	2.4
Anosmia	8	0.3
Asymptomatic	74	2.7

The mortality rate during the study period was 4.8% (132 deaths out of 2744 patients). The median age of the patients who died was 73.5 years, with the minimum age being 37 years and the maximum being 100 years. The majority of the patients who did not survive were above 60 years of age (86.36%). The profile of mortality among the patients is shown in Table 3. The remaining 2612 patients were discharged following the state guidelines.

**Table 3:** Mortality profile of patients (n=132).

	Frequency	Percentage
Gender		
Males	77	58.33
Females	55	41.67
Age		
<60 Years	18	13.64
>60 Years	114	86.36

## Discussion

The present study was conducted in a tertiary care center in north Kerala, India, with a total of 2744 COVID-19 patients admitted between May and October 2021. The mean age of the study participants was 53.33±18.45 years. This was much higher than the mean age seen in other similar studies in Kerala. In a study conducted in Thrissur by Thomas et al., the mean age was found to be 35.8 years, and in another study in Trivandrum, it was 36.5±13.9 years [7,8]. In other studies conducted in India and around the world [9-13], the mean age was also found to be less than that in the present study (35.9±14.7 years to 46.16±13.7 years). This difference in age may be explained by the fact that most of these studies were conducted in the beginning of the pandemic, when expatriates (who are likely to belong to the younger working class) accounted for the majority of the cases, whereas the present study was conducted after community transmission was well established.

The proportion of males and females in the present study was comparable with the percentage of females being slightly higher than males (51.5% as compared to 48.5%). In contrast, other studies in Kerala [7,8] had a male preponderance similar to several other studies in India [11,12,14]. In similar studies conducted in Nigeria and Kuwait, the proportion of males was also higher [9,15]. Comparable male and female proportions were seen in studies conducted in China [16,17].

The major symptoms reported in the present study were fever, cough, and breathlessness. More than 40% of the participants presented with fever and cough. Headache, sore throat, fatigue, anosmia, rhinitis/nasal congestion, gastrointestinal symptoms like diarrhea, nausea, and vomiting, and chest discomfort and myalgia were also reported. About 74 (2.7%) study participants were asymptomatic. In similar studies conducted in Kerala, fever and cough were the major presenting complaints, as seen in the present study [7,8]. Though fever and cough were the major symptoms in other studies conducted in India, the proportion of asymptomatic patients was much higher than that in the present study [11,12,14].

In studies carried out in China, the symptoms reported by most of the patients were fever and cough [16,17], whereas in a study in Nigeria, the major symptoms were breathlessness and fatigue, followed by fever and cough [9]. In a study conducted in multiple centers in Europe [13], the major symptoms were headache (70.3%) and anosmia (70.2%), whereas in the present study, only 19.2% had headaches, and anosmia was reported by only 0.3%. In a meta-analysis by Grant et al. [18] with 148 studies and more than 24,000 participants, it was found that fever and cough were the major symptoms, as seen in the present study also.

The mortality rate in the present study was found to be 4.8% (132 deaths), with the majority of deaths occurring in the elderly (>60 years). The mortality rate is high compared to the national and state averages, which are 1.18% and 1.05%, respectively; this may be due to the fact that the present study included only hospitalized patients [1,2]. In a study conducted in Kerala [7], the mortality rate was comparable (3.2%) to that in the present study, whereas in another regional study [8], it was found to be much lower (0.99%). In a study by Sherwal et al. [14], the mortality rate was 3.6%, while in studies conducted elsewhere in India, the rates were found to be lower [10,11]. In a meta-analysis conducted in China [19], the mortality rate was found to be 3.6%, which is close to the rate in the present study. Table 4 gives a comparison of the present study with similar studies.

**Table 4:** Comparison of the present study with other similar studies.

	Present study (n=2744)	Thomas et al. (n=32) [7]	Varghese et al. (n=202) [8]	Mohan et al. (n=144) [11]	Sherwal et al. (n=308) [14]	Gupta et al. (n=21) [12]	Otuonye et al. (n=154) [9]	Guan et al. (n=1099) [17]	Lechien et al. (n=1420) [13]
Study period	May-October 2021	March 2020	March 2020	March-April 2020	March-May 2020	February-March 2020	May-August 2020	January 2020	March-April 2020
Study setting	Kerala, India	Kerala, India	Kerala, India	New Delhi, India	New Delhi, India	New Delhi, India	Nigeria	China	Multicentric study, Europe
Age (years)	53.33±18.45	35.8	36.5±13.9	40.1±13.1	48	40.3	46.16±13.7	47	39.17±12.09
Males (%)	48.5	62.5	80.2	93.1	77.6	66.7	75	58.1	-
Symptoms (%)									
Fever	44.56	54.2	45	17.4	39	42.9	45.5	88.7	45.4
Cough	41.1	37.5	25	34.7	38.6	42.9	40.3	67.8	63.2
Breathlessness	29.5	37.5	4.5	5.6	12.7	4.8	48.1	18.7	-
Fatigue	21.8	-	7	1.4	-	-	48.7	38.1	-
Headache	19.2	20.8	14	1.4	5.2	13.6	-	13.6	70.3
GI symptoms	14.5	25	-	2.8	-	-	-	-	-
Myalgia	10.4	20.8	7.5	3.5	14	-		14.9	62.5
Sore throat	5.9	8.3	22	21.5	13.3	23.8	13	13.9	-
Chest discomfort	5.6	-	-	0.7	-	-	33.1	-	-
Rhinitis	2.4	-	12.5	21.5	9.4	-	7.8	4.8	60.1
Anosmia	0.3	8.3	12	-	1.3	-	13	-	70.2
Asymptomatic	2.7	25	19.3	44.4	30.8	42.9	10.4	-	-
Mortality									
Case fatality rate	4.8	3.1	0.99	1.4	3.6	-	2.6	1.4	-

Limitations

The study did not include clinical or laboratory parameters. Since the data was reported from a tertiary care hospital, it may not be representative of the general population.

## Conclusions

The present study highlights the symptomatology and other demographic features of COVID-19 patients admitted to a hospital in Kerala, India, in comparison with other regional, national, and international studies. We have included 2744 patients in the study and the main symptoms reported were fever, cough, and breathlessness. The mortality rate was found to be higher than state and national averages possibly due to the fact that the study was conducted in a tertiary care center; it was also higher in patients more than 60 years.

The study had limitations, such as not including more demographic details or clinical and laboratory parameters, yet it provides valuable insights into the local impact of COVID-19 and may guide future public health policies and interventions aimed at mitigating the spread of the virus. As COVID-19 symptomatology keeps evolving with mutations in the virus and the effect of widespread vaccination, the present study can provide a baseline for further research into the disease.
